# Meloxicam Targets COX-2/NOX1/NOX4/Nrf2 Axis to Ameliorate the Depression-like Neuropathology Induced by Chronic Restraint Stress in Rats

**DOI:** 10.3390/ph16060848

**Published:** 2023-06-06

**Authors:** Hany H. Arab, Ali Khames, Mostafa K. Mohammad, Shuruq E. Alsufyani, Ahmed M. Ashour, Azza A. K. El-Sheikh, Hany W. Darwish, Amany M. Gad

**Affiliations:** 1 Department of Pharmacology and Toxicology, College of Pharmacy, Taif University, P.O. Box 11099, Taif 21944, Saudi Arabia; s.alsofyani@tu.edu.sa; 2 Department of Biochemistry, Faculty of Pharmacy, Cairo University, Cairo 11562, Egypt; 3 Department of Pharmacology and Toxicology, Faculty of Pharmacy, Sohag University, Sohag 82511, Egypt; ali.khames@pharm.sohag.edu.eg; 4 Department of Pharmacology and Toxicology, Faculty of Pharmacy, Sphinx University, New Assiut City 71515, Assiut, Egypt; mk8882000@gmail.com; 5 Department of Pharmacology and Toxicology, College of Pharmacy, Umm Al Qura University, P.O. Box 13578, Makkah 21955, Saudi Arabia; amashour@uqu.edu.sa; 6 Basic Health Sciences Department, College of Medicine, Princess Nourah bint Abdulrahman University, P.O. Box 84428, Riyadh 11671, Saudi Arabia; aaelsheikh@pnu.edu.sa; 7 Department of Pharmaceutical Chemistry, College of Pharmacy, King Saud University, Riyadh 11451, Saudi Arabia; hdarwish@ksu.edu.sa; 8 Department of Pharmacology and Toxicology, Faculty of Pharmacy, Sinai University, Kantara Branch, Ismailia 41636, Egypt; amany.gad@su.edu.eg; 9 Department of Pharmacology, Egyptian Drug Authority (EDA)—Formerly NODCAR, Giza 12654, Egypt

**Keywords:** meloxicam, stress, neuroinflammation, NOX1, NOX4, Nrf2

## Abstract

Meloxicam has shown significant neuroprotection in experimental models of stroke, Alzheimer’s disease, and Parkinson’s disease. However, the potential of meloxicam to treat depression-like neuropathology in a chronic restraint stress (CRS) model and the associated molecular changes has been insufficiently explored. The current work aimed to explore the potential neuroprotective actions of meloxicam against CRS-evoked depression in rats. In the current experiments, animals received meloxicam (10 mg/kg/day; i.p.) for 21 days, and CRS was instigated by restraining the animals for 6 h/day during the same period. The sucrose preference test and the forced swimming test were used to explore the depression-linked anhedonia/despair, whereas the open-field test examined the animals’ locomotor activity. The current findings revealed that CRS elicited typical depression behavioral anomalies in the animals, including anhedonia, despair, and diminished locomotor activity; these findings were reinforced with Z-normalization scores. These observations were corroborated by brain histopathological changes and increased damage scores. In CRS-exposed animals, serum corticosterone spiked, and the hippocampi revealed decreased monoamine neurotransmitter levels (norepinephrine, serotonin, and dopamine). Mechanistically, neuroinflammation was evident in stressed animals, as shown by elevated hippocampal TNF-α and IL-1β cytokines. Moreover, the hippocampal COX-2/PGE_2_ axis was activated in the rats, confirming the escalation of neuroinflammatory events. In tandem, the pro-oxidant milieu was augmented, as seen by increased hippocampal 8-hydroxy-2′-deoxyguanosine alongside increased protein expression of the pro-oxidants NOX1 and NOX4 in the hippocampi of stressed animals. In addition, the antioxidant/cytoprotective Nrf2/HO-1 cascade was dampened, as evidenced by the lowered hippocampal protein expression of Nrf2 and HO-1 signals. Interestingly, meloxicam administration mitigated depression manifestations and brain histopathological anomalies in the rats. These beneficial effects were elicited by meloxicam’s ability to counteract the corticosterone spike and hippocampal neurotransmitter decrease while also inhibiting COX-2/NOX1/NOX4 axis and stimulating Nrf2/HO-1 antioxidant pathway. Together, the present findings prove the neuroprotective/antidepressant actions of meloxicam in CRS-induced depression by ameliorating hippocampal neuroinflammation and pro-oxidant changes, likely by modulating COX-2/NOX1/NOX4/Nrf2 axis.

## 1. Introduction

Major depressive disorder (MDD) is the most common neuropsychiatric disorder, and it has several serious consequences, including suicidal attempts [1]. The pathogenesis of MDD comprises increased stimulation of the hypothalamic–pituitary–adrenal (HPA) pathway. The neuropathology of depression also involves the depletion of norepinephrine, serotonin, and dopamine in the brain’s hippocampus [2,3]. Ample evidence reveals that stress is a crucial risk factor for MDD incidence. Different stress models have been characterized in rodents to resemble the human manifestations of depression, such as anhedonia, despair, and dampened locomotor activity [2,4]. Among them, the chronic restraint stress (CRS) paradigm has been described to develop typical depression manifestations [4]. In this model, the animals are restrained for 6 h/day for 21 days, culminating in neurobehavioral aberrations analogous to the human condition. These include excessive HPA hyperactivity, resulting in escalating levels of corticosterone, the main glucocorticoid in rodents [5]. Hence, the CRS paradigm has been regarded as a common model for investigating the efficacy of agents with potential antidepressant features [4].

Similar to human depression, the neuropathology of the CRS paradigm implicates aberrant activation of the HPA axis, resulting in escalating levels of plasma corticosterone in animals [2,6]. Consequently, multiple brain regions, including the hippocampus, experience excessive neuroinflammatory reactions and overshooting of the pro-inflammatory cytokines, e.g., interleukin 1 beta (IL-1β) and tumor necrosis factor-alpha (TNF-α) [2,3,7,8,9]. Excessive pro-inflammatory cytokine production has been reported to activate the transcription of cyclo-oxygenase 2 (COX-2), the rate-limiting enzyme in prostaglandin E_2_ (PGE_2_) production, thereby intensifying neuroinflammation [10]. Together, these events instigate hippocampal neuronal loss and disrupted functionality, culminating in depression-associated behavioral anomalies [4].

In addition to neuroinflammation, oxidative stress has been implicated in the neuropathogenesis of depression. In this context, hippocampal overproduction of 8-hydroxy-2′-deoxyguanosine (8-OHdG) has been reported in rodents challenged with CRS [11]. In the context of the redox milieu, inhibition of the antioxidant nuclear factor erythroid 2-related factor-2 (Nrf2)/heme oxygenase 1 (HO-1) cascade has been characterized in CRS paradigms [12]. In fact, Nrf2 depletion has been tightly linked to depression in humans and experimental animals. Interestingly, the upregulation of Nrf2 by antidepressant modalities has been seen as a successful tool for combating depression [13]. However, caution should be applied for augmenting Nrf2 levels, since Nrf2 has displayed tumorigenic and atherogenic potential [13,14]. In the same regard, upregulated protein expression of the pro-oxidant NADPH oxidase 1 (NOX1) has been previously described in chronic social defeat stress and corticosterone-evoked depression-like behavior in mice [15]. Moreover, the crucial role of NADPH oxidase has been described in the pathogenesis of chronic neurodegenerative disorders, including Alzheimer’s disease and Parkinson’s disease, as well as ischemic/traumatic brain damage [16]. However, the potential role of NADPH oxidase in CRS-induced depression-like behavior has been inadequately explored.

In the clinical setting, serious side effects have been associated with the intake of current mainstay antidepressant therapy, particularly when used for extended durations or in large doses [17]. Due to the multifactorial etiology of MDD, the currently available tricyclic antidepressants and selective serotonin reuptake inhibitors fail to elicit remission in one-third of depression patients [18]. Thus, it remains imperative to search for alternative modalities with potential antidepressant actions. The neuropathogenesis of depression has revealed that overexpression of COX-2 in the hippocampus is tightly linked to susceptibility to stress-induced anhedonia in rodents, a hallmark of depression manifestations that is characterized by a diminished response to pleasure/rewarding stimuli [19]. Under stressful events, the protein expression of COX-2 is upregulated in the hippocampal cornu ammonis 1 (CA1) and dentate gyrus regions, culminating in neuronal death in these hippocampal areas [20]. Under pathological states, COX-2 overexpression is associated with increased PGE_2_ synthesis, an event that enhances HPA axis activity and triggers a surge in pro-inflammatory cytokines, culminating in MDD symptoms [21]. Notably, the COX-2 inhibition approach in MDD patients has been reported to normalize aberrant cortisol production [22]. However, controversial reports have described that genetic ablation of COX-2 demonstrated an increased incidence of neuronal damage, microglia/astrocyte activation, and excessive production of pro-inflammatory signals [23]. Hence, further exploration of the role of COX-2 inhibition is still warranted.

With COX-2 inhibition ranging from 3 to 77 times that of COX-1, meloxicam has been identified as a preferential COX-2 inhibitor [24]. Classically, it has demonstrated promising results in the management of postoperative pain and inflammation [25]. The ability of meloxicam to gain access to brain tissue has been reported by crossing the blood–brain barrier [26,27], especially under neuropathological conditions [28,29]. Interestingly, meloxicam showed remarkable neuroprotection in experimental models of focal ischemia [30] and stroke [25]. In experimental stroke, the neuroprotective effects of meloxicam were mediated by the modulation of glial scar reactivity and increased axonal sprouting. Meloxicam also counteracted neuronal injury and cognitive impairment in diabetic rats via inhibition of the hippocampal COX-2/PGE_2_ pathway and dampening of pro-inflammatory cytokine production [28]. Likewise, meloxicam rescued scopolamine-evoked Alzheimer-like manifestations by dampening oxidative stress and enhancing antioxidant signals [29]. In a rodent model of Parkinson’s disease, meloxicam improved motor dysfunction and dopaminergic neurodegeneration by activating protein kinase B (AKT) signaling, favoring neuronal survival [31]. Meanwhile, the ability of meloxicam to prevent the onset of pain after nerve root compression has been demonstrated by inhibiting the stimulation of microglia and astrocytes and dampening the pro-oxidant and pro-inflammatory signals in the spinal cord and dorsal root ganglia [32]. In an experimental model of postsurgical mood disorder, meloxicam attenuated neuroinflammation and anhedonia in mice [10]. However, it failed to affect depressive-like behavior in human immunodeficiency virus (HIV)-transgenic female rats [33]. Thus, the ameliorative effect of meloxicam on depression warrants further investigation. In this context, the potential of meloxicam to mitigate CRS-evoked depression manifestations has not been addressed in previous studies. Therefore, the aim of the current study was to determine whether meloxicam could mitigate the depression driven by CRS and the underlying processes, with a specific emphasis on hippocampal neuroinflammation and pro-oxidant signals. Of note, the current study focused on behavioral outcomes and hippocampal changes in animals. In perspective, the hippocampus has been characterized as the brain structure that plays a crucial role in determining the vulnerability or resilience of an individual to stress-evoked depression and other mental health issues [19,34].

## 2. Results

### 2.1. Meloxicam Improves CRS-Evoked Depression-like Behavior

**Figure 1:**
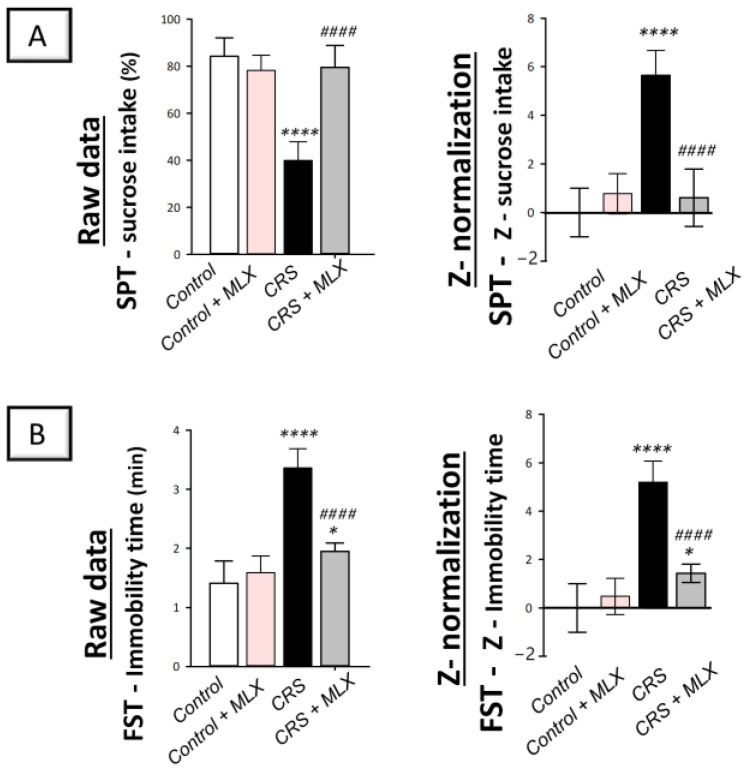
Impact of meloxicam on the depression manifestations evoked by chronic restraint stress (CRS) in animals. Herein, the depression manifestations were improved by meloxicam in the sucrose preference test (SPT) and forced swimming test (FST). The higher sucrose intake percentage in the SPT (**A**) and diminished immobility time (**B**) in the FST were indicators of meloxicam’s beneficial outcomes. Calculation of the Z-values is detailed in Section 4.5.5 (Materials and Methods). Bars express mean ± standard deviation (*n* = 6). **** p < 0.0001 or * p < 0.05 vs. control; #### *p* < 0.0001 vs. CRS (one-way ANOVA and Tukey’s multiple comparison test). CRS, chronic restraint stress; MLX, meloxicam.

Examination of depression manifestations elicited by CRS was conducted using the sucrose preference test (SPT) and forced swimming test (FST) in rats. In perspective, the SPT examines anhedonia, a diminished response to rewarding stimuli, such as sucrose drinking, as a hallmark of depression [19,35]. In the same regard, the FST investigates the despair component of depression [36]. As depicted in Figure 1A,B (left panels), animals that underwent CRS displayed significant group differences in the sucrose consumption percentage [F (3, 20) = 40.17, *p* < 0.0001], as reported by one-way ANOVA in the SPT. Likewise, significant group differences were displayed in immobility time [F (3, 20) = 54.09, *p* < 0.0001], as reported by one-way ANOVA in the FST. When compared to the control animals, CRS significantly decreased the sucrose consumption percentage by 52.6% (*p* < 0.0001, Tukey post hoc test) in the SPT, revealing behavioral despair. In the FST, CRS significantly increased immobility time by 138.3% (*p* < 0.0001, Tukey post hoc test). Favorably, the behavioral anomalies in the CRS-exposed rats were improved by the administration of meloxicam (10 mg/kg; i.p.), as shown by a significant elevation in the sucrose consumption percentage of 98.8% (*p* < 0.0001, Tukey post hoc test) compared to the CRS group in the SPT. Moreover, meloxicam significantly lowered immobility time by 42% (*p* < 0.0001, Tukey post hoc test) compared to the CRS group in the FST.

Z-score normalization, a calculation method that produces strong and trustworthy values for comparison [20], reinforced meloxicam’s beneficial effects against depression manifestations. In perspective, the raw data are transformed into the number of standard deviations from the control’s mean using this calculation method. As depicted in Figure 1A,B (right panels), CRS resulted in significant changes in the Z-values of sucrose consumption by 566% (*p* < 0.0001, Tukey post hoc test) in the SPT and immobility time by 519.9% (*p* < 0.0001, Tukey post hoc test) in the FST when compared to control rats. Meloxicam administration to the CRS-challenged animals significantly reversed these changes. Together, these data demonstrate meloxicam’s ability to improve CRS-evoked depression manifestations in animals.

### 2.2. Meloxicam Counteracts CRS-Induced Decline in Movement Activity

**Figure 2:**
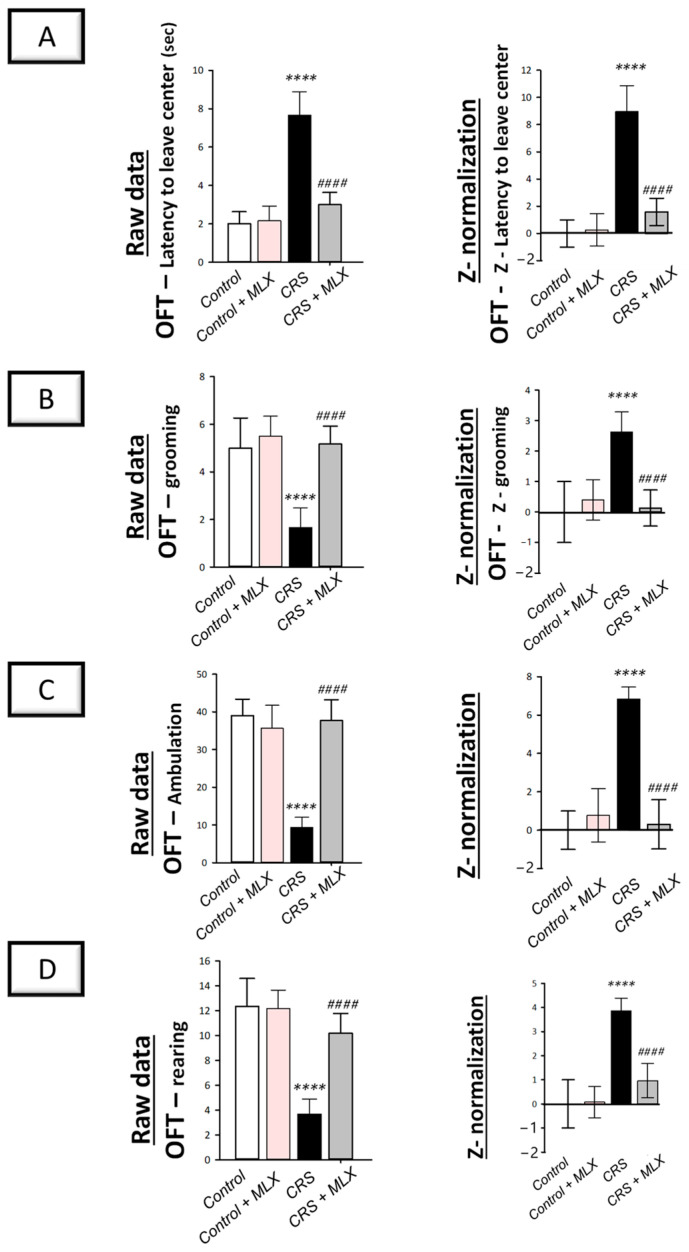
Impact of meloxicam on the depression-linked locomotor activity decline that was evoked by chronic restraint stress (CRS) in rats. Herein, meloxicam improved the depression-linked decrease in locomotor activity in the open-field test (OFT). This was reflected by the lowered latency time to leave the center (**A**) alongside increased grooming (**B**), ambulation (**C**), and rearing (**D**) in the OFT. Calculation of the Z-values is detailed in Section 4.5.5 (Materials and Methods). Bars express mean ± standard deviation (*n* = 6). **** p < 0.0001 vs. control; #### *p* < 0.0001 vs. CRS (one-way ANOVA and Tukey’s multiple comparison test). CRS, chronic restraint stress; MLX, meloxicam.

The open-field test (OFT) in rats, which examines the locomotor activity of animals [2,37], was applied to examine the movement activity decline linked to depression. As depicted in Figure 2 (left panels), animals that underwent CRS displayed significant group differences in latency to leave the central square [F (3, 20) = 60.61, *p* < 0.0001] and grooming [F (3, 20) = 21.76, *p* < 0.0001], as reported by one-way ANOVA in the OFT. Likewise, one-way ANOVA revealed significant group differences in ambulation [F (3, 20) = 51.58, *p* < 0.0001] and rearing [F (3, 20) = 35.21, *p* < 0.0001]. When compared to the control animals, CRS significantly increased latency time (*p* < 0.0001, Tukey post hoc test) by 283.3% and significantly decreased grooming, ambulation, and rearing (*p* < 0.0001, Tukey post hoc test) by 66.7%, 76.1%, and 70.3%, respectively, revealing behavioral despair. Compared to the CRS group, meloxicam improved the decline in locomotor activity by significantly lowering latency time by 60.9% (*p* < 0.0001, Tukey post hoc test) and significantly increasing grooming, ambulation, and rearing (*p* < 0.0001, Tukey post hoc test) by 210%, 303.6%, and 177.3%, respectively.

Z-score normalization reinforced meloxicam’s beneficial effects against the CRS-triggered decline in locomotor activity. As depicted in Figure 2 (right panels), CRS resulted in significant changes (*p* < 0.0001, Tukey post hoc test) in the Z-values for latency time, grooming, ambulation, and rearing by 895.9%, 263.5%, 684.2%, and 385%, respectively, when compared to the control rats. Meloxicam administration to the CRS-challenged animals significantly reversed these changes. Together, these data demonstrate meloxicam’s ability to improve the CRS-evoked depression-linked locomotor activity decline in animals.

### 2.3. Meloxicam Attenuates CRS-Induced Histopathological Aberrations in the Brain Cortex and Hippocampus

**Figure 3:**
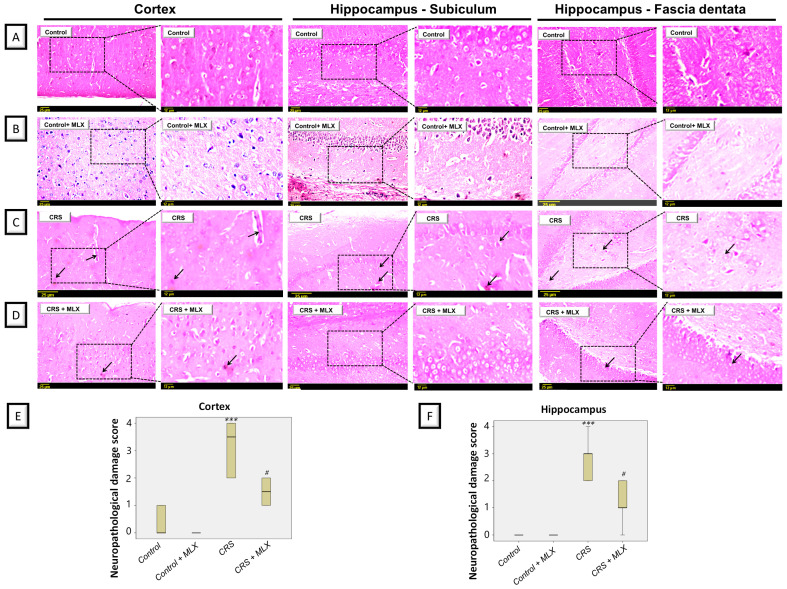
Impact of meloxicam on nuclear pyknosis and neuronal degeneration in the cortices and hippocampi of animals challenged with chronic restraint stress (CRS). Hematoxylin and eosin (H&E) was applied to stain the brain sections, and light microscopy was performed for inspection (representative pics; scale bars = 25 µm and 12 µm). Herein, attenuation of CRS-triggered nuclear pyknosis and neuronal degeneration was driven by meloxicam administration. (**A**,**B**) An intact histological structure was observed in the cerebral cortex alongside the hippocampal subiculum and fascia dentata in the control and meloxicam-treated groups. (**C**) Marked nuclear pyknosis (arrow) and neuronal degeneration were observed in the brain cortices and hippocampi in the CRS group. (**D**) Meloxicam administration to the CRS-exposed group revealed fewer incidences of nuclear pyknosis and neuronal degeneration. (**E**,**F**) Quantification of cortical and hippocampal nuclear pyknosis, respectively, confirmed the ability of meloxicam to dampen brain pathological changes. The favorable effects of meloxicam were seen by the lowered neuropathological damage scores in these brain areas. Data are median/interquartile range (*n* = 6). *** p < 0.001 vs. control; # *p* < 0.05 vs. CRS (Kruskal–Wallis and Dunn’s post-test). CRS, chronic restraint stress; MLX, meloxicam.

Data from rodent models of stress-triggered depression have revealed remarkable degenerative aberrations in the cortices and hippocampi of animals [22,23]. Accordingly, the potential ameliorative impact of meloxicam was further examined by histopathology in the cortices and hippocampi of animals subjected to CRS. As depicted in Figure 3, CRS resulted in evident cortical and hippocampal histopathological changes in the animals. This was reflected by marked pyknosis and neuronal degeneration in these brain areas. Quantification of the nuclear pyknosis in the cortex and hippocampus was described as the neuropathological damage scores (Figure 3E,F). Kruskal–Wallis analysis revealed significant group differences in damage scores in the cortex [H (3, 20) = 18.72, *p* = 0.0003] and hippocampus [H (3, 20) = 17.71, *p* = 0.0005]. Meloxicam significantly dampened the elevated damage scores in both brain regions. These findings show the ability of meloxicam to ameliorate brain histopathological alterations in CRS-exposed animals.

### 2.4. Meloxicam Lowers CRS-Induced Spike in Serum Corticosterone

**Figure 4:**
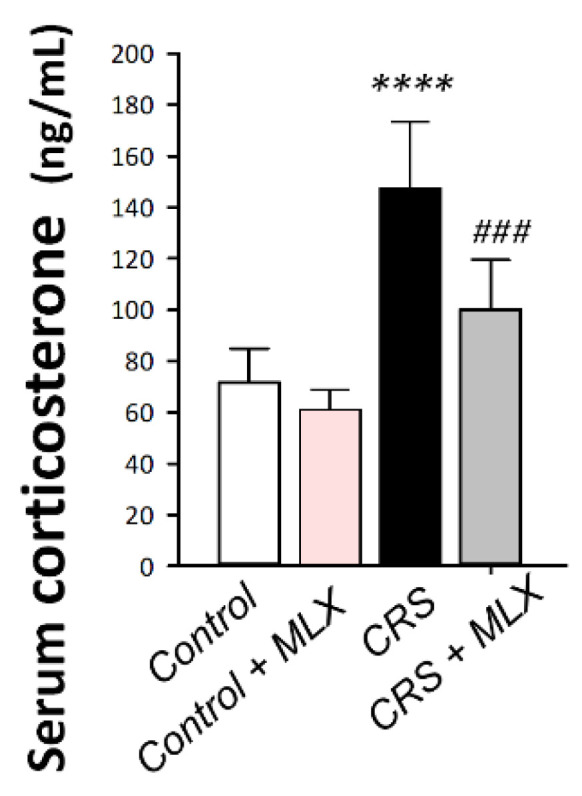
Impact of meloxicam on serum corticosterone in animals challenged with chronic restraint stress (CRS). Meloxicam diminished the serum corticosterone spike. Bars express mean ± standard deviation (*n* = 6). **** p < 0.0001 vs. control; ### *p* < 0.001 vs. CRS (one-way ANOVA and Tukey’s multiple comparison test). CRS, chronic restraint stress; MLX, meloxicam.

To characterize HPA axis activity, corticosterone serum levels were measured as the chief glucocorticoid in rats [21]. As depicted in Figure 4, one-way ANOVA revealed significant group differences in serum corticosterone [F (3, 20) = 29.41, *p* < 0.0001]. When compared to the control animals, CRS significantly increased serum corticosterone by 106.4% (*p* < 0.0001, Tukey post hoc test). Compared to the CRS group, meloxicam curbed the stress response by significantly lowering serum corticosterone by 32.1% (*p* < 0.001, Tukey post hoc test). This finding demonstrates meloxicam’s ability to curtail the stress response that was prompted by CRS in animals by diminishing serum corticosterone.

### 2.5. Meloxicam Counteracts CRS-Induced Decline in Hippocampal Monoamine Neurotransmitters

**Figure 5:**
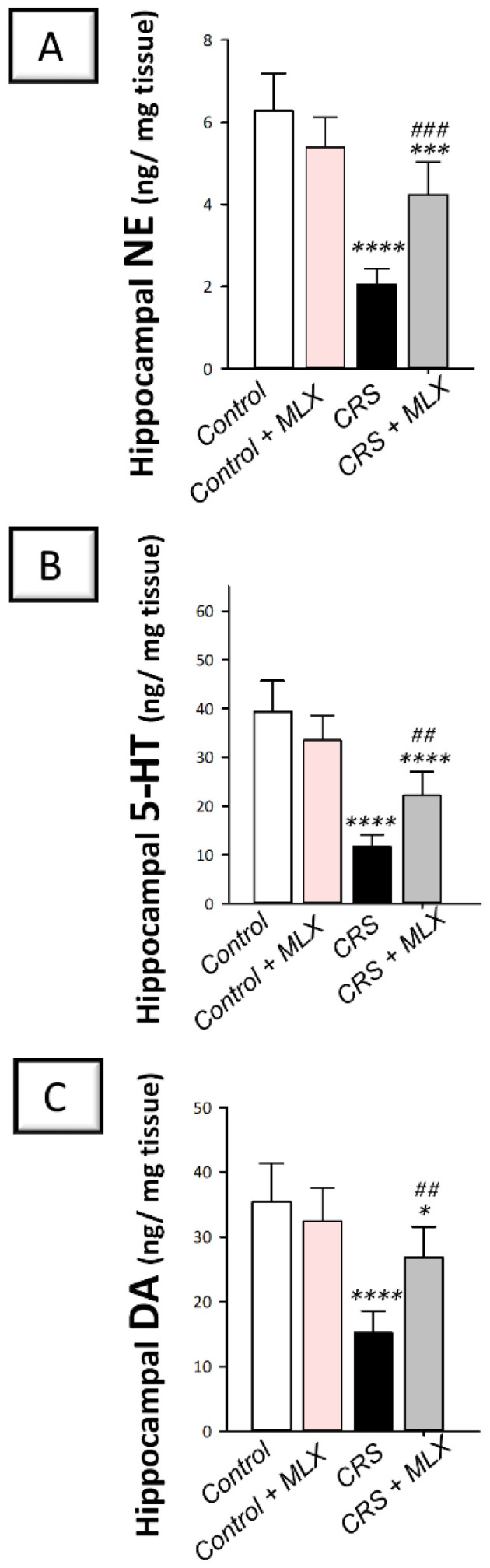
Impact of meloxicam on hippocampal monoamine neurotransmitter levels in animals challenged with chronic restraint stress (CRS). Augmentation of hippocampal levels of norepinephrine (**A**), serotonin (**B**), and dopamine (**C**) was triggered by meloxicam administration in rats. Bars express mean ± standard deviation (*n* = 6). **** p < 0.0001, *** p < 0.001, or * p < 0.05 vs. control; ### *p* < 0.001 or ## *p* < 0.01 vs. CRS (one-way ANOVA and Tukey’s multiple comparison test). CRS, chronic restraint stress; MLX, meloxicam.

As depicted in Figure 5A–C, one-way ANOVA revealed significant group differences in hippocampal norepinephrine [F (3, 20) = 36.85, *p* < 0.0001], serotonin [F (3, 20) = 38.0, *p* < 0.0001], and dopamine [F (3, 20) = 19.66, *p* < 0.05]. CRS significantly diminished (*p* < 0.0001, Tukey post hoc test) the hippocampal content of norepinephrine (NE) by 67.3%, serotonin (5-HT) by 70.3%, and dopamine (DA) by 57% when compared to the control animals. Compared to the CRS group, meloxicam augmented the hippocampal content of the three neurotransmitters by significantly elevating NE, 5-HT, and DA by 106.6% (*p* < 0.001, Tukey post hoc test), 89.5% (*p* < 0.01, Tukey post hoc test), and 76.2% (*p* < 0.01, Tukey post hoc test), respectively. These data show that meloxicam’s ability to enhance the aforementioned hippocampal neurotransmitters is, at least partly, implicated in the observed amelioration of depression manifestations in animals.

### 2.6. Meloxicam Suppresses Hippocampal NOX1/NOX4 Pro-Oxidant Signals and Enhances the Antioxidant Nrf2/HO-1 Pathway in Rats Exposed to CRS

**Figure 6:**
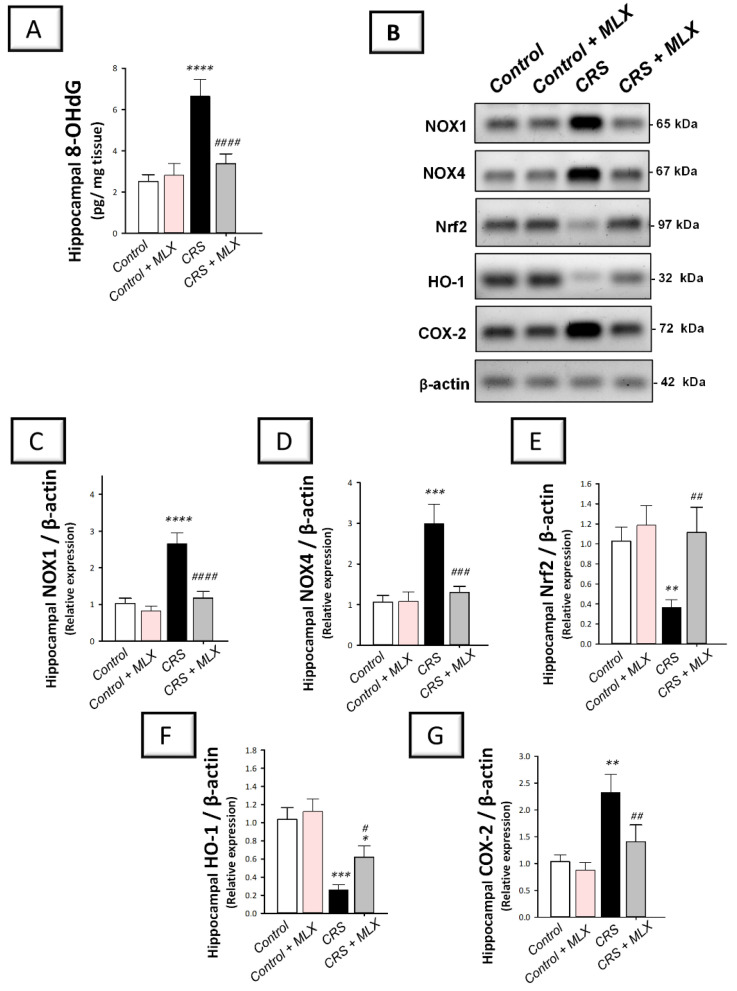
Effect of meloxicam on hippocampal NOX1/NOX4/Nrf2/COX2 pathway in animals challenged with chronic restraint stress (CRS). Suppression of hippocampal 8-hydroxy-2′-deoxyguanosine (8-OHdG; (**A**)), NADPH oxidase 1 (NOX1; (**C**)), and NADPH oxidase 4 (NOX4; (**D**)) was triggered by meloxicam administration in rats exposed to CRS. Moreover, meloxicam augmented the hippocampal levels of nuclear factor erythroid 2-related factor 2 (Nrf2; (**E**)) and heme oxygenase 1 (HO-1; (**F**)) in rats exposed to CRS. In tandem, meloxicam suppressed the hippocampal protein expression of cyclo-oxygenase 2 (COX-2; (**G**)). The immunoblots demonstrate the protein expression of the signals implicated in NOX1/NOX4/Nrf2/COX2 pathway (**B**). Bars express mean ± standard deviation (*n* = 6 for 8-OHdG; *n* = 3 independent values for immunoblotting data). **** p < 0.0001, *** p < 0.001, ** p < 0.01, or * p < 0.05 vs. control; #### *p* < 0.0001, ### *p* < 0.001, ## *p* < 0.01, or # *p* < 0.05 vs. CRS (one-way ANOVA and Tukey’s multiple comparison test). CRS, chronic restraint stress; MLX, meloxicam.

The hippocampal pro-oxidant milieu of animals was investigated by measuring 8-OHdG levels. In addition, the protein expression of two isoforms of the pro-oxidant NADPH oxidases 1 and 4 (NOX1 and NOX4) was investigated with Western blotting. In the same context, the antioxidant Nrf2/HO-1 pathway was explored by measuring the protein levels of Nrf2 and HO-1 antioxidant signals [12,13,38] in the hippocampi of animals. As depicted in Figure 6A–D, one-way ANOVA revealed significant group differences in the hippocampal content of 8-OHdG [F (3, 20) = 66.32, *p* < 0.0001], NOX1 [F (3, 8) = 54.17, *p* < 0.0001], and NOX4 [F (3, 8) = 31.35, *p* < 0.001]. When compared to the control animals, CRS significantly increased (*p* < 0.0001, Tukey post hoc test) hippocampal 8-OHdG, NOX1, and NOX4 by 164.3%, 157.9%, and 181.2%, respectively. Compared to the CRS group, meloxicam suppressed hippocampal pro-oxidant signals by significantly diminishing 8-OHdG, NOX1, and NOX4 by 49.3% (*p* < 0.0001, Tukey post hoc test), 55.8% (*p* < 0.0001, Tukey post hoc test), and 56.7% (*p* < 0.001, Tukey post hoc test), respectively.

Regarding the antioxidant Nrf2/HO-1 pathway, one-way ANOVA revealed significant group differences in the hippocampal levels of Nrf2 [F (3, 8) = 13.35, *p* = 0.0018] and HO-1 [F (3, 8) = 35.03, *p* < 0.0001], as illustrated in Figure 6E,F. When compared to the control animals, CRS significantly diminished the hippocampal protein expression of Nrf2 and HO-1 by 64.3% (*p* < 0.01, Tukey post hoc test) and 75% (*p* < 0.001, Tukey post hoc test), respectively. Compared to the CRS group, meloxicam augmented the hippocampal content of Nrf2 and HO-1 by 204.3% (*p* < 0.01, Tukey post hoc test) and 138.8% (*p* < 0.05, Tukey post hoc test), respectively. These data demonstrate that meloxicam’s ability to suppress hippocampal NOX1/NOX4 pro-oxidant signals and enhance the antioxidant Nrf2/HO-1 pathway is, at least partly, implicated in the mitigation of depression manifestations in animals.

### 2.7. Meloxicam Inhibits Hippocampal Pro-Inflammatory Signals and COX-2/PGE_2_ in Rats Exposed to CRS

**Figure 7:**
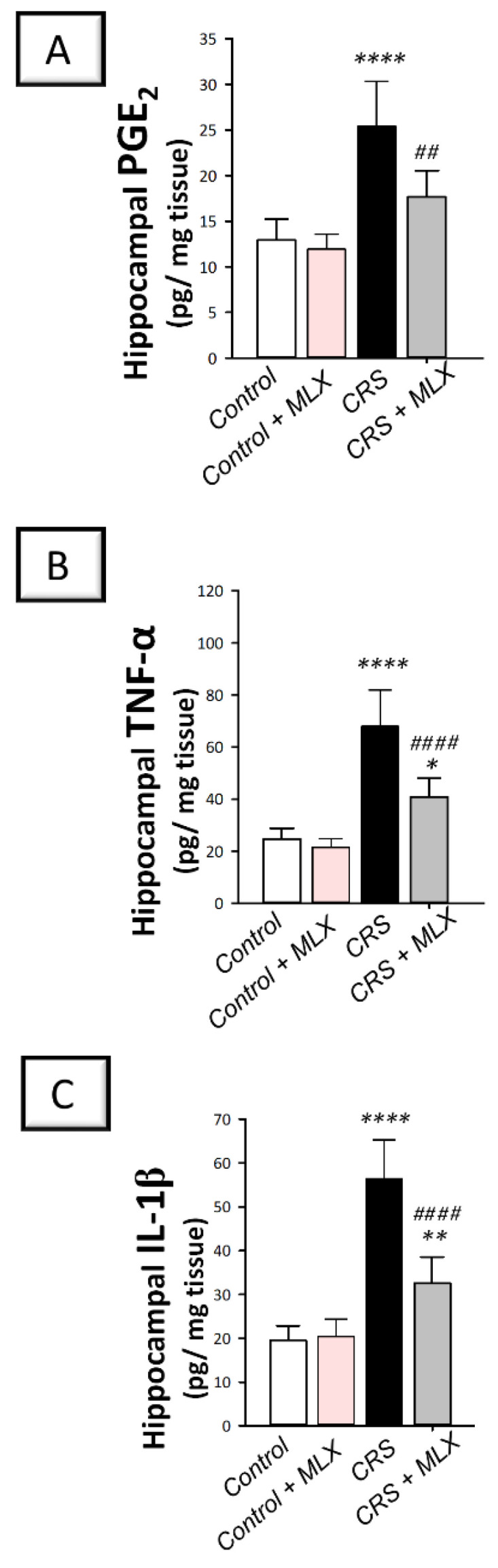
Effect of meloxicam on hippocampal PGE_2_ and pro-inflammatory cytokines in animals challenged with chronic restraint stress (CRS). Herein, meloxicam administration to CRS-insulted animals suppressed the levels of hippocampal prostaglandin E2 (PGE_2_; (**A**)), tumor necrosis factor-alpha (TNF-α; (**B**)), and interleukin 1 beta (IL-1β; (**C**)). Bars express mean ± standard deviation (*n* = 6). **** p < 0.0001, ** p < 0.01, or * p < 0.05 vs. control; #### *p* < 0.0001 or ## *p* < 0.01 vs. CRS (One-way ANOVA and Tukey’s multiple comparison test). CRS, chronic restraint stress; MLX, meloxicam.

The hippocampal pro-inflammatory milieu of animals was investigated by measuring the levels of pro-inflammatory PGE_2_, TNF-α, and IL-1β [2,3,9,39]. Moreover, COX-2 protein expression was examined [19,40] with Western blotting. As depicted in Figure 7A–C, one-way ANOVA revealed significant group differences in the hippocampal content of PGE_2_ [F (3, 20) = 22.51, *p* < 0.0001], TNF-α [F (3, 20) = 40.06, *p* < 0.0001], and IL-1β [F (3, 20) = 50.23, *p* < 0.0001] alongside COX-2 protein expression [F (3, 8) = 20.17, *p* < 0.01] seen in Figure 6G. When compared to the control animals, CRS significantly elevated hippocampal PGE_2_, TNF-α, and IL-1β (*p* < 0.0001, Tukey post hoc test) alongside COX-2 protein expression (*p* < 0.01, Tukey post hoc test) by 96.6%, 176.1%, 189.3%, and 125.1%, respectively. Compared to the CRS group, meloxicam diminished these pro-inflammatory events by significantly lowering PGE_2_, TNF-α, and IL-1β levels alongside COX-2 protein expression by 30.4% (*p* < 0.01, Tukey post hoc test), 39.9% (*p* < 0.0001, Tukey post hoc test), 42.3% (*p* < 0.0001, Tukey post hoc test), and 39.5% (*p* < 0.01, Tukey post hoc test), respectively. These data depict that meloxicam’s ability to lower hippocampal pro-inflammatory signals and COX-2/PGE_2_ pathway is, at least partly, implicated in the attenuation of depression manifestations in animals.

## 3. Discussion

**Figure 8:**
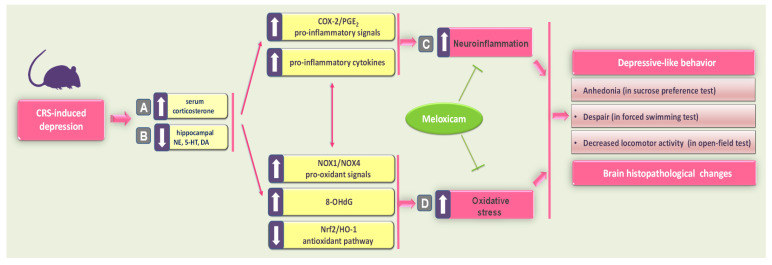
A summary of the mechanisms that mediated meloxicam’s antidepressant ability to counteract CRS-induced depression. Exposure of animals to CRS triggered a circulating corticosterone spike (**A**) with concomitant depletion of monoamine neurotransmitters (norepinephrine, serotonin, and dopamine) in the hippocampi of rats (**B**). (**C**) These events instigated hippocampal neuroinflammation, as seen by the upregulated protein expression of COX-2 alongside increased hippocampal PGE_2_ and pro-inflammatory cytokines. (**D**) In tandem, CRS resulted in an excessive hippocampal pro-oxidant milieu, as seen by increased 8-OHdG, upregulated expression of NOX1 and NOX4, and inhibition of the antioxidant Nrf2/HO-1 pathway. Together, these pro-inflammatory/pro-oxidant events instigated hippocampal histopathological changes and typical depressive behavioral changes, including anhedonia, behavioral despair, and locomotor deficits. Interestingly, meloxicam administration inhibited hippocampal neuroinflammation and pro-oxidant events by inhibiting COX-2/NOX1/NOX4 cascade and stimulating the Nrf2/HO-1 pathway. Concertedly, these molecular changes resulted in an enhancement of hippocampal neurotransmitters and an attenuation of depressive-like behavior in rats. The solid arrow indicates stimulation, while the blunt arrow indicates inhibition. CRS, chronic restraint stress; COX-2, cyclo-oxygenase 2; DA, dopamine; HO-1, heme oxygenase 1; 5-HT, serotonin; NE, norepinephrine; Nrf2, nuclear factor erythroid 2-related factor-2; NOX1, NADPH oxidase 1; NOX4, NADPH oxidase 4; 8-OHdG, 8-hydroxy-2′-deoxyguanosine; PGE_2,_ prostaglandin E_2_.

In the present report, meloxicam administration improved CRS-induced depression-like manifestations, including anhedonia and behavioral despair, as seen by elevated sucrose consumption in the SPT and diminished immobility time in the FST. Meanwhile, meloxicam counteracted the CRS-induced decline in locomotor activity in the OFT. These favorable outcomes were driven by multipronged mechanisms involving attenuation of neuroinflammation and pro-oxidative events, likely by modulating COX-2/NOX1/NOX4/Nrf2 axis in the hippocampi of animals (Figure 8). In fact, the observed multipronged targets addressed by meloxicam may support its beneficial effects on depression, a neurological disorder with a multifactorial pathogenesis [15,19,41,42].

The neuropathogenesis of depression involves multiple theories that describe enhanced activity in the HPA axis, culminating in enhanced glucocorticoid levels in the systemic circulation [2,3,4,19]. Evidence also exists that the depletion of hippocampal monoamine neurotransmitters is a hallmark in the neuropathology of depression [43]. Animal models of stress-induced depression-like manifestations also replicate aberrant activation of the HPA axis alongside a decline in hippocampal serotonin, norepinephrine, and dopamine neurotransmitters [2,3,11]. In the context of depression symptoms, the depression manifestations that develop in rodents are analogous to the human condition. In perspective, exposure to CRS precipitates anhedonia—a decreased responsiveness to rewarding stimuli seen by diminished sucrose intake by animals—alongside decreased locomotor activity [2,3,19]. In this context, hippocampal overexpression of COX-2 has been characterized as a crucial determinant of the susceptibility of mice to stress-evoked anhedonia in a chronic mild stress paradigm. In perspective, only anhedonic (stress-susceptible) but not non-anhedonic (stress-resilient) animals demonstrated hippocampal COX-2 overexpression, confirming the critical role of COX-2 expression in determining individual susceptibility to stress [19]. Consistent with these studies, the current findings revealed that CRS resulted in typical depression-like behavioral anomalies, including anhedonia, evidenced by lowered sucrose consumption in the SPT, behavioral despair, as seen by increased immobility time in the FST, and lowered movement activity in the OFT. These depressive behavioral deficits are driven by increased stress and the associated spike in circulating corticosterone; these observations were proven herein and in previous reports [2,3,11]. The interplay between stress and hippocampal monoamine neurotransmitter decline has been previously characterized [2,6]. In perspective, stress-evoked neurotransmitter depletion in the hippocampus is attributed to accelerated monoamine breakdown and/or reduced synthesis in response to the stress-triggered death of monoamine neurons in the hippocampus [44]. Interestingly, meloxicam attenuated the serum corticosterone spike and enhanced the content of serotonin, norepinephrine, and dopamine in the hippocampus, thereby promoting improved behavioral outcomes. In fact, previous studies have revealed the efficacy of several modalities—including monoamine oxidase inhibitors (MAOIs) and selective serotonin reuptake inhibitors (SSRIs)—that can enhance the production of monoamine neurotransmitters in the brain hippocampus as successful tools for improving depression symptoms [2,3,4,11,12]. Of note, pharmacological COX-2 inhibition with celecoxib has previously demonstrated marked antidepressant effects against stress-evoked anhedonia, a crucial hallmark of depression [19]. Consistent with the observed elevation in hippocampal neurotransmitters, meloxicam has previously been reported to counteract striatal dopamine depletion in a rodent model of 1-methyl-4-phenyl-1,2,3,6-tetrahydropyridine (MPTP)-induced Parkinson’s disease [45].

Ample evidence has revealed that CRS provokes enhanced neuroinflammation in several brain areas, including the hippocampus [2,3,4,11,12], the brain region that determines an individual’s predisposition to MDD [19,34,46]. Essentially, microglia and astrocytes express an increased content of pro-inflammatory cytokines, promoting neuronal hyperexcitability [47]. Evidence has also shown that CRS intensifies pro-inflammatory cytokine production in the hippocampus [3,4]. Notably, overexpression of pro-inflammatory cytokines and COX-2 has been reported in the hippocampi of depressive syndrome-susceptible animals but not in the stress-resilient cohort [19], and these experimental findings coincide with the clinical data of depressed patients [42]. Moreover, overproduction of PGE_2_ has been reported in the hippocampi of experimental animals in a chronic mild stress model [41] and in the plasma and cerebrospinal fluid of depressed patients [19]. In keeping with these studies, the present work revealed that CRS triggered an exaggerated hippocampal pro-inflammatory status, evidenced by increased TNF-α and IL-1β alongside COX-2 overexpression and intensified PGE_2_ levels in stressed animals. The current findings and the previous literature [19] pinpoint the role of neuroinflammation and COX-2 overexpression in increasing an individual’s predisposition to MDD. Under pathological conditions, overexpression of COX-2 instigates the synthesis of PGE_2_, which, in turn, activates the HPA axis and provokes a surge in pro-inflammatory signals, culminating in MDD symptoms [21]. This pro-inflammatory cytokine surge and exaggerated neuroinflammation disrupt hippocampal serotonin transmission, culminating in depressive-like “sickness behavior” [19,21]. Notably, the hippocampal overexpression of COX-2 has been linked to neuronal death in the CA1 and dentate gyrus areas of the hippocampus [20]. In a rodent model of chronic mild stress, COX-2 overexpression and a PGE_2_ spike in the dendritic spines of the hippocampus have been proven to boost oxidative stress, dendritic plasticity, and depressive-like behaviors [41]. Herein, meloxicam curbed the pro-inflammatory events by lowering hippocampal pro-inflammatory cytokines and inhibiting COX-2/PGE_2_ pathway in animals challenged with CRS. Essentially, abrogation of TNF-α and IL-6 alongside COX-2 downregulation mediated the neuroprotective effects of meloxicam in experimental LPS-evoked brain neuroinflammation [48], quinolinic acid-induced Huntington’s disease-like manifestations [49], and in a painful nerve root injury model [32]. In rodent models of cognitive impairment in diabetic rats [28] and pentylenetetrazol-induced experimental epilepsy, meloxicam showed notable neuroprotection and rescued the behavioral deficits by dampening TNF-α and interleukins together with COX-2/PGE_2_ pathway [50]. Likewise, dampening neuroinflammation and glial scar reactivity mediated the neuroprotective role of meloxicam in a transient cerebral ischemia model, culminating in improved behavioral neurological deficits in rats [25]. In the same regard, a previous report by Strekalova et al. [19] revealed the efficacy of COX-2 inhibition with celecoxib in ameliorating depression-linked anhedonia in a chronic mild stress paradigm in mice. Relevant to targeting depression neuropathology with COX-2 inhibitors, pharmacological inhibition and genetic ablation of COX-1 have been proven to attenuate depressive syndrome [19]. However, COX-2 selective/preferential inhibition would display better compliance since the COX-1 inhibition approach is tightly linked to significant adverse effects, such as gastric and duodenal ulcers [19,24]. Of note, current findings on meloxicam’s attenuation of neuroinflammation and depression manifestations are not entirely consistent with the data that have reported the ability of meloxicam to dampen the inflammatory response but failed to dampen depressive-like behavior in HIV-transgenic female rats [33]. This controversy might be related to differences in the animal species (wild type vs. transgenic), the nature of the experimental model, and the severity of depression symptoms. The marked anti-inflammatory features of meloxicam may support its efficacy, at least as an adjunct modality for depression. This approach may be particularly crucial in MDD patients with high baseline pro-inflammatory markers. In perspective, patients with high baseline C-reactive protein (CRP) displayed more aggressive symptoms of depression, including little physical activity, bad mood, suicidality, and cognitive deficits [19]. In line with this notion, COX-2 positron emission tomography (PET) imaging—a tool for monitoring in vivo COX-2 upregulation—is an in-progress avenue for the identification of MDD subgroups with active neuroinflammation [51]. Hence, the use of agents that can suppress neuroinflammation would pave the way for more advanced personalized management of depression.

Mounting evidence reveals that CRS provokes excessive pro-oxidant events in several brain regions, including the hippocampus, in rodents [2,3,8]. The enhanced vulnerability of the brain to oxidation is attributed to its elevated metabolic demands [16]. Oxidative stress has been involved in the neuropathology of depression in human subjects and animal models [15]. In perspective, exposure to chronic stress triggers a surge in neuronal ROS production alongside derangement of the antioxidant system [15]. NADPH oxidase, a microglia-derived pro-oxidant enzyme, is a superoxide-generating flavoenzyme whose primary function is the generation of ROS [52]. The ROS generated by NOX trigger mitochondrial dysfunction, which generates an additional surge in ROS, culminating in a vicious cycle of oxidative stress [16,52]. Interestingly, the expression of NOX1 and NOX4 has been identified in several brain regions, including the hippocampus [16]. Rodent models of chronic social defeat stress and corticosterone-evoked depressive-like behavior have revealed upregulated protein expression of the pro-oxidant NOX1 in the mesoprefrontal projection in the brain of animals [15]. In the context of pro-oxidant insult, NOX1 upregulation and increased DNA oxidative damage were reported herein (the hippocampal spike of 8-OHdG) and in previous models of stress-induced depression [41]. Moreover, NOX4 is the only NOX subtype whose main product is H_2_O_2_, which has a longer half-life and enhanced tissue permeability than the classical NOX product superoxide anion [16,52]. Evidence exists for the upregulation of NOX4 in experimental models of stress-induced depression [41] and other neurological disorders, such as intracerebral hemorrhage [15]. The interplay between NOXs and neuroinflammation has been characterized, where the upregulation of NOX1 and NOX4 is enhanced by a variety of inflammatory signals, including TNF-α and interleukins, culminating in brain injury [16,41,52]. Together, in response to persistent stress, the enhanced hippocampal pro-oxidant and pro-inflammatory responses provide a positive feedback loop that excessively activates the HPA axis, resulting in hippocampal neuronal loss and depression manifestations [4]. In accordance with these studies, the current study’s findings demonstrated increased levels of 8-OHdG, overexpression of the pro-oxidants NOX1 and NOX4, and inhibition of Nrf2/HO-1 pathway in the hippocampi of animals challenged with CRS. Essentially, neuronal oxidative stress is associated with intensified circulating glucocorticoids, which instigate enhanced ROS production [3]. Of note, therapeutic strategies that target NOX1 and NOX4 downregulation have been envisioned as successful tools for the attenuation of depression [15,41] and other neurodegenerative diseases [52]. In perspective, NOX1 genetic ablation ameliorated the depressive-like behavioral hallmarks in rodent models of chronic social defeat stress and corticosterone-evoked depression [15]. Likewise, NOX4 knockdown enhanced neuronal tolerance to oxidative stress, curbed ROS production, and dampened neuronal cell death in a rodent model of stroke [52]. Consistent with these data, the current study characterized meloxicam’s antioxidant effects by diminishing 8-OHdG, downregulating the pro-oxidants NOX1 and NOX4, and activating the antioxidant/cytoprotective Nrf2/HO-1 pathway in the hippocampi of rats with CRS-evoked depression, resulting in improved depressive behavioral outcomes, including attenuated anhedonia and despair. These data are in keeping with previous reports that characterized the antioxidant potential of meloxicam by diminishing lipid peroxides and replenishing neuronal antioxidant defenses in an experimental model of scopolamine-induced cognitive deficit [29], pentylenetetrazol-kindled mice [50], and in a painful nerve root injury model [32]. A previous report by Song et al. [41] suggested that oxidative events may mediate the impact of COX-2 overexpression in the hippocampi of animals on the development of depression-associated behavioral deficits, including anhedonia. This was evidenced by the ameliorative effect of the antioxidant N-acetylcysteine on COX-2 overexpression, oxidative stress markers, dendritic spine deficiency, and depressive behavior in Wistar rats. Moreover, the pathology of neurological disorders revealed that only targeting ROS scavenging demonstrated minimal success in clinical trials [16]. Interestingly, shifting the focus to the inhibition of ROS generation by dampening NOX1 and NOX4 is likely a more successful avenue for therapy [15,16,41].

## 4. Materials and Methods

### 4.1. Chemicals

Meloxicam was procured from Boehringer Ingelheim International GmbH (Ingelheim, Rhein, Germany). All chemicals were obtained as having the highest analytical grade quality.

### 4.2. Animals

The current study was performed using adult male Wistar albino rats weighing 200–250 g. The animal house of the Egyptian Drug Authority (EDA, Giza, Egypt) provided the animals needed for the experimental work. Before the start of the experimental protocol, animal adaptation was applied for 10 days. Under conventional laboratory settings (21–24 °C temperature, 40–60% relative humidity, and 12/12 h diurnal/nocturnal cycle), all animals received free access to standard laboratory food and drinking water. Approval of the current set of experiments was obtained from the Research Ethics Committee for Experimental and Clinical Studies at the EDA under permit number NODCAR/I/44/2022. The rats were handled carefully, as instructed by the guidelines of ARRIVE and the U.K. Animals Act, 1986.

### 4.3. Experimental Protocol

**Figure 9:**
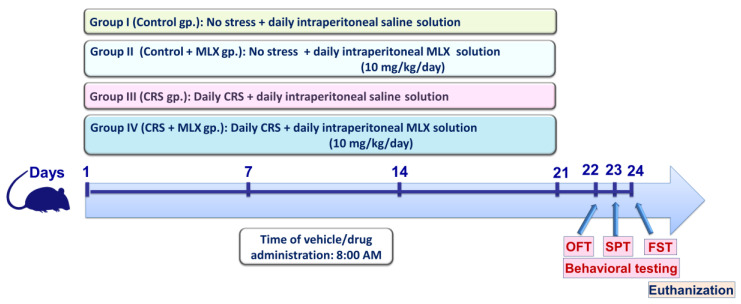
Schematic presentation of the current study protocol. Behavioral testing was conducted at the conclusion of the experimental period in the following order, commencing with the least stressful test: (1) OFT, (2) SPT, and (3) FST, respectively, as previously reported [11,53]. CRS, chronic restraint stress; FST, forced swimming test; MLX, meloxicam; OFT, open-field test; SPT, sucrose preference test.

Forty adult male Wistar rats were assigned randomly into four experimental groups in the current study (*n* = 10 rats/group). Figure 9 illustrates a summary of the current experimental protocol. Rats in group 1 (control group) received a saline intraperitoneal injection every day for 3 weeks without being subjected to stress. Rats in group 2 (control + MLX group) received 10 mg/kg/day of meloxicam intraperitoneally for 3 weeks without being subjected to stress. Rats in group 3 (the chronic restraint stress (CRS) group) underwent a 3-week period of daily intraperitoneal saline administration alongside daily exposure to 6 h of restraining during the 21-day period. Rats in group 4 (CRS + MLX group) underwent a 3-week period of daily intraperitoneal meloxicam administration (10 mg/kg/day) alongside daily exposure to 6 h of restraining during the 21-day period. Of note, the administration of the vehicles/drugs took place at 8:00 A.M. During the investigation, the identity of the samples was blinded to the observer. Moreover, an independent technician conducted the sample coding/decoding procedures.

The chosen dose of meloxicam is in keeping with previous studies that have demonstrated its neuroprotective efficacy in rodent models of Parkinson’s disease [31], scopolamine-induced Alzheimer-like neuropathology and cognitive impairment [29], and postoperative mood disorder [10]. Moreover, the present CRS regimen is in line with several reports [2,41,42].

After the completion of the study protocol, the behavioral testing was applied on days 22–24, starting from the least to most stressful behavioral tests [11,53], as illustrated in Figure 9. Then, animal anesthesia (sodium pentobarbital, 50 mg/kg; i.p.) was applied [54], and an intracardiac puncture was performed to collect blood for serum isolation. Animal sacrifice was conducted by decapitation, and immediate isolation of brain hippocampi was performed. The hippocampi were kept at −80 °C for the assays. For ELISA and Western blotting, homogenization of the hippocampus was performed in a RIPA buffer/protease inhibitor cocktail [55]. For histopathology, 4 brains from 4 random animals per experimental group were dissected out and fixed in 10% formalin saline.

### 4.4. Procedures of the CRS

With the aid of restraining tubes with dimensions of 9 cm × 24 cm × 6 cm, the animals underwent daily exposure to CRS for 6 h per day for 3 consecutive weeks. During the diurnal phase, the CRS was applied at random times. In this regard, movement restriction of the animal’s head and limbs was attained by modifying the restraining tube length. Notably, animals in the control and control + MLX groups did not undergo stress exposure, as they were left in a separate room without contact with the stressed rats.

### 4.5. Behavioral Outcomes

#### 4.5.1. Sucrose Preference Test (SPT)

An examination of anhedonia, a hallmark behavior of depression in which the animal suffers from a diminished response to pleasure/rewarding stimuli, such as drinking sucrose solution [19,35], was performed using the SPT [35]. For the acclimatization of the animals, they were given 2 similar 1% sucrose solution bottles to drink exclusively from for 72 h. Before starting the SPT, animal deprivation from the drinking solution was applied for 16 h, and then the SPT was started. In the SPT, the animal was individually housed in a cage and given a free choice to drink from 2 bottles of either 1% sucrose solution (100 g—weight) or water (100 g—weight). At the start and end of the test period, weighing of the 2 bottles was performed, and hence the sucrose consumption was calculated. On the testing day, the SPT was initiated when the animal’s activity phase (dark cycle) started. The side preference in drinking behavior was avoided by changing the place of the 2 bottles every 6 h. Of note, spillage of the drinking liquids during the SPT was reduced by pre-filling the bottles and holding them upside-down for at least 12 h before the test. Moreover, balancing the temperature between the drinking fluid and the ambient air temperature was performed by keeping the drinking bottles in the same room [19]. This prevented the leakage of fluids from the bottles due to the temperature difference between the drinking fluid and the ambient room temperature. Calculation of the sucrose preference percentage was performed as previously characterized [56] = consumption of sucrose solution (g) × 100/(consumption of sucrose solution (g) + consumption of water (g)) [56].

#### 4.5.2. Forced Swimming Test (FST)

This test was used to explore depression-linked despair according to the method established by Posolt et al. [36]. At 25 °C, animals were placed in a clear cylindrical tank (40 cm in height × 22 cm in diameter) filled with water to the 25 cm mark. Herein, the animals were allowed to swim for 5 min, and the “swimming time” taken by each animal to reach the wall of the tank was noted. In addition, the time span during which the animal did not swim was calculated as the “immobility time”. To sidestep potential confusing data due to odor cues, the water was replaced at the end of each trial.

#### 4.5.3. Open-Field Test (OFT)

An examination of the animals’ locomotor activity was performed with the aid of the OFT [2,37]. The OFT is a wooden box split into 25 identical squares (dimensions: 60 × 60 × 20 cm). Herein, the animal was placed at the central square of the OFT apparatus, and the animal’s behavior was tracked for 3 min in a quiet room under dim white light. The time taken by each rat to start a movement was defined as the latency time to leave the central square during the 3 min span. During the same period, ambulation, grooming, and rearing were recorded. After each individual trial, ethyl alcohol solution (70%) was used to clean the wooden box to avoid confusing results due to odor cues.

#### 4.5.4. Sequence of the Behavioral Tests

As illustrated in Figure 9, the behavioral tests were conducted with a specific sequence in which the animals were subjected to the least stressful behavioral test first, followed by the more stressful tests [11,53]. Hence, the sequence of the behavioral tests was (1) OFT, (2) SPT, and (3) FST (days 22–24, respectively).

#### 4.5.5. Normalization of Z-Scores

For the behavioral tests, normalization of Z-scores was applied to create a more reliable calculation to express the obtained data values for the effect of meloxicam on the depression-associated endpoints in the 3 behavioral tests (SPT, FST, and OFT) [57]. In perspective, this transforms the raw data of all the study groups into the number of standard deviations from the control’s mean. The following equation was used to calculate the Z-scores: Z = X − µ/σ. Herein, “X” represents the individual raw data value, “µ” stands for the control group’s mean, and “σ” stands for the control group’s standard deviation.

### 4.6. Histopathology and Neuropathological Scoring

Brain tissue specimens were preserved for 24 h in 10% neutral buffered formalin and processed for paraffin embedding [58]. Coronal brain slices (4 µm) were cut to show the hippocampal areas in the specimens. For general morphological inspection, the sections were stained with hematoxylin and eosin (H–E) and observed with light microscopy (Leica Microsystems GmbH, Ernst-Leitz-Strasse, Wetzlar, Germany). Herein, the number of specimens from each experimental group was 4, and the selection was conducted randomly to avoid bias.

In the cortex and hippocampus, scoring of nuclear pyknosis was applied and characterized as neuropathological damage scores based on a 0–4 scoring system [53,59]. In brief, a score of 4 indicated that nuclear pyknosis was observed in more than 60% of the affected area, a score of 3 indicated that nuclear pyknosis was observed in 40–60% of the affected area, a score of 2 indicated that nuclear pyknosis was observed in 10–40% of the affected area, a score of 1 indicated that nuclear pyknosis was observed in less than 10% of the affected area, and a score of 0 indicated a lack of a specific lesion.

### 4.7. Serum Corticosterone

The blood samples were collected into serum-separating tubes and allowed to clot for 2 h at 25 °C. For serum separation, centrifugation of the test tubes was performed at 1000× *g* for 15 min. To assess corticosterone levels, a rat-specific corticosterone ELISA kit was used (Cat. no. ER0859, Wuhan Fine Biotech., Wuhan, China), under the guidance of the vendor’s recommendations. The optical density measurement of the final color was applied at 450 nm.

### 4.8. Hippocampal Neurotransmitters

The hippocampal homogenate supernatants were used for the assay of norepinephrine (NE), serotonin (5-HT), and dopamine (DA). To this end, specific ELISA kits were utilized for the measurement of these monoamine neurotransmitters [60]. For DA (Cat. no. MBS7214676), for 5-HT (Cat. no. MBS2700308, MyBioSource Inc., San Diego, CA, USA), and for NE (Cat. no. LS-F28027, LifeSpan Biosciences, Inc., Seattle, WA, USA), the assays were performed as instructed by the vendors. The optical density measurement of the final color was applied at 450 nm.

### 4.9. Measurement of 8-OHdG

The hippocampal homogenate supernatants were used to assay 8-hydroxy-2′-deoxyguanosine (8-OHdG). To this end, a rat-specific ELISA kit was used (Cat. no. MBS267513, MyBioSource, San Diego, CA, USA), under the guidance of the vendor’s recommendations. The optical density measurement of the final color was applied at 450 nm.

### 4.10. Immunoblotting

Examination of NOX1, NOX4, Nrf2, HO-1, and COX-2 was performed by an immunoblotting protocol, as previously reported [61,62]. To this end, homogenization of the hippocampus was conducted in an ice-cold lysis solution (Tris-HCL pH 8.0, 1% NP40 (1% *v*/*v*), 0.1% SDS, and 0.5% sodium deoxycholate; provided with phenylmethylsulfonylfluoride (PMSF)). Centrifugation of the lysates was conducted at 10,000× *g*, and the supernatants were isolated. Equal protein extracts (50 μg) were resolved with SDS-PAGE. Transfer of the resolved proteins was performed with a semi-dry electro-blotter into a nitrocellulose membrane. Membrane blockade with 5% non-fat milk/TBST was performed for 1 h, and membrane incubation with the appropriate primary antibody was applied overnight in a fridge. To this end, incubation was performed using specific antibodies against NOX-1 (Cat. no. ab131088; dilution 1:2000), NOX4 (Cat. no. ab154244, Abcam, Waltham, MA, USA; dilution 1: 2000), Nrf2 (Cat. no. 33649, Cell Signaling Technology, Beverly, MA, USA; dilution 1:1000), HO-1 (Cat. no. ab154244; dilution 1:2000), and COX-2 (Cat. no. ab179800, Abcam, Waltham, MA, USA; dilution 1:3000). Membrane washing was performed, and secondary antibody incubation was conducted for 2 h at room temperature (Cell Signaling Technology, Beverly, MA, USA; dilution 1:10,000). Detection of the protein bands was performed using an enhanced chemiluminescence kit (Cat. no. RPN2132, ECL plus; GE Healthcare, Buckinghamshire, UK). Molecular Analyst Software (Bio-Rad, Hercules, CA, USA) was utilized for protein band quantification.

### 4.11. Pro-Inflammatory Signals

The hippocampal homogenate supernatants were used for the assay of PGE_2_, IL-1β, and TNF-α. To this end, rat-specific ELISA kits were utilized, as follows: (Cat. no. MBS262150) for PGE_2_, (Cat. no. MBS825017) for IL-1β, and (Cat. no. MBS355371, MyBioSource, San Diego, CA, USA) for TNF-α, as instructed by the vendors. The optical density measurement of the final color was applied at 450 nm.

### 4.12. Statistical Analysis

The data were analyzed with GraphPad Prism software (GraphPad Prism Inc., San Diego, CA, USA). Initially, data normality was checked with the Shapiro–Wilk test. For normally distributed values, one-way ANOVA and the post hoc Tukey’s post-test were used for data analysis. For the neuropathological damage scores, the Kruskal–Wallis test and Dunn’s post-test were used, since these values are non-parametric. The threshold for statistical significance was set at *p* < 0.05.

## 5. Conclusions

Together, the favorable actions of meloxicam in attenuating CRS-induced depression manifestations, including anhedonia and despair, were driven by multipronged mechanisms. Principally, the suppression of hippocampal neuroinflammation and pro-oxidant signals by modulating COX-2/NOX1/NOX4/Nrf2 axis was crucial in the observed neuroprotection. Notably, the witnessed antidepressant features of meloxicam may support its efficacy, at least as an adjunct modality for MDD, particularly MDD associated with high baseline pro-inflammatory markers. Accordingly, suppression of the neuroinflammation component of depression may pave the way for more advanced personalized depression management [51,63]. However, supplemental studies are needed to examine the efficacy of meloxicam in the clinical setting. Additional studies are also needed to delineate the detailed molecular mechanisms of meloxicam’s neuroprotection in CRS-induced depression.

## Data Availability

Data are contained within the article.
